# Wise Prescriptions: Prevalence and Predictors of Polypharmacy in Patients with Type 2 Diabetes Mellitus in Primary Care: A Retrospective Cross-Sectional Study

**DOI:** 10.3390/jcm15083002

**Published:** 2026-04-15

**Authors:** Mohammed M. Alsultan, Danya R. Al Thani, Sara A. Shwaiheen, Ethabah A. Al Drees, Mohammed A. Al Drees, Reem D. AlQahtani, Amnah A. Alnubi, Shuaa Y. Alali, Amani M. AlQarni

**Affiliations:** 1 Department of Pharmacy Practice, College of Pharmacy, Imam Abdulrahman Bin Faisal University, Dammam 34212, Saudi Arabia; 2 Family and Community Medicine Department, College of Medicine, King Fahd Hospital of the University, Imam Abdulrahman Bin Faisal University, Dammam 31441, Saudi Arabia

**Keywords:** polypharmacy, hyperpolypharmacy, diabetes mellitus, type 2 diabetes mellitus, multimorbidity, primary care

## Abstract

**Background/Objectives**: Diabetes mellitus is a common chronic disease that may lead to multimorbidity and high drug use. Therefore, this study aims to examine the prevalence of polypharmacy and hyperpolypharmacy among adult patients diagnosed with type 2 diabetes mellitus (T2DM) with its associated factors. **Methods**: This is a retrospective cross-sectional study conducted from 1 May 2023 to 31 October 2024. The outcomes in our study were polypharmacy (from five to nine drugs) and hyperpolypharmacy (≥10 drugs). Baseline and demographic characteristics, along with multinomial logistic regression, were used to analyze the data. **Results**: The total number of patients with T2DM was 2435. The prevalence rate of polypharmacy was 46.98%, while hyperpolypharmacy was 24.27%. Older age was significantly associated with a higher risk of polypharmacy [OR = 1.031, 95% (1.022–1.040)] and hyperpolypharmacy [OR = 1.037, 95% (1.026–1.049)]. In addition, patients with higher levels of hemoglobin A1c showed a significantly higher risk of polypharmacy and hyperpolypharmacy ([OR = 1.162, 95% (1.105–1.221)] and [OR = 1.284, 95% (1.209–1.364)], respectively). The comorbidities that increased the odds of hyperpolypharmacy were hypertension [OR = 2.136, 95% (1.449–3.148)], pulmonary disease [OR = 2.375, 95% (1.292–4.367)], mental disorders [OR = 6.269; 95% (3.284–11.964], and congestive heart failure [OR = 8.014, 95% (2.768–23.200)]. **Conclusions**: The prevalence of polypharmacy and hyperpolypharmacy is high in patients with T2DM. The predictors that may play a significant role in increasing the risk of hyperpolypharmacy are the poor control of HbA1c and the coexistence of comorbidities. Providing proper prescribing of patients’ therapy plans can improve individuals’ health outcomes. Therefore, this study highlights the important role of primary care physicians in coordinating care, along with clinical pharmacists, in the identification of polypharmacy.

## 1. Introduction

Diabetes mellitus (DM) is a highly prevalent chronic disease that is rising at an exponential rate worldwide. The International Diabetes Federation estimates that the number of people affected by DM will increase from 589 million in 2024 to 853 million by 2050 worldwide [1]. The prevalence of DM in the Middle East and North Africa (MENA) region was 17.6% in 2024. From the MENA region, Saudi Arabia is the fourth top country regarding the number of people affected by DM, with an estimated 5.3 million cases. In addition, the prevalence of DM in Saudi Arabia was reported as 23.1% in 2024 for ages 20–79 years, and it is expected to reach 25.4% in 2050 [2,3].

The development of DM is complex and multifactorial, including environmental and genetic factors. DM is a long-lasting metabolic illness that happens when the body cannot successfully use insulin and/or the pancreas fails to produce enough insulin, resulting in hyperglycemia. Thus, if hyperglycemia is not effectively controlled, it can lead over time to both macrovascular and microvascular complications [4].

Polypharmacy is a negative health consequence that is commonly encountered in the diabetic population. Therefore, polypharmacy refers to issues associated with multiple-drug consumption and the regular use of five or more medications [5,6,7,8,9]. Diabetic patients have a higher risk of polypharmacy since most of the patients have coexisting chronic medical conditions, such as hypertension (HTN), dyslipidemia, cardiovascular-related disorders, and mental health conditions. All of this will result in the need for different medications, making the management of polypharmacy more challenging [10,11,12,13,14,15]. Furthermore, the assessment of polypharmacy in clinic settings is important due to the significant risk that may occur. Consequently, the possible clinical effects were drug–drug interactions, adverse events, and quality of life [16,17,18].

Although there is a high prevalence of DM in Saudi Arabia, it represents that there is a lack of research regarding the prevalence of polypharmacy and its risk factors for individuals with type 2 diabetes mellitus (T2DM), specifically in the Eastern Province of Saudi Arabia. Therefore, the aim of our study is to examine the prevalence of polypharmacy and hyperpolypharmacy among patients diagnosed with T2DM with its associated factors. Therefore, the significance of this study could lead to a better understanding of factors that may lead to a better achievement of safer and more effective therapeutic approaches for T2DM patients.

## 2. Materials and Methods

### 2.1. Study Design

The design was a retrospective cross-sectional study that lasted from 1 May 2023 to 31 October 2024.

### 2.2. Study Population and Data Source

The study population was adults (≥18 years) diagnosed with T2DM, and data were retrieved from the electronic medical records (EMRs) of the primary care clinic of King Fahad University Hospital, Al-Khobar, Saudi Arabia. The data were comprehensive, which included the demographic characteristics (age, gender, and nationality) and the lab values (hemoglobin A1c (HbA1c), albumin, and serum creatinine). In addition, there were comorbidity files (HTN, cancer, mental disorders, pulmonary diseases, neurological diseases, congestive heart failure (CHF), and dyslipidemia) and a drugs file.

### 2.3. Study Outcomes

The outcome in our study was the number of chronic medications that have been used by patients (no polypharmacy, polypharmacy, and hyperpolypharmacy). The definition of each category was stated based on no polypharmacy (<5 drugs), polypharmacy (from 5 to 9), and hyperpolypharmacy (≥10) [9]. The over-the-counter drugs were not included.

### 2.4. Risk Factors

The risk factors in our study were demographic characteristics of patients, such as age, gender, and nationality. The comorbidities file includes HTN, cancer, mental disorders, pulmonary diseases, neurological disease, CHF, and dyslipidemia. The lab values for each patient include HbA1c, albumin, and estimated glomerular filtration rate (eGFR). The estimated glomerular filtration rate (eGFR) in mL/min/1.73 m^2^ was calculated based on the chronic kidney disease epidemiology (CKD-EPI) collaboration formula [19].

### 2.5. Ethical Consideration

This study was approved by the Institutional Review Board of the Imam Abdulrahman Bin Faisal University, Dammam, Saudi Arabia (IRB-2024-05-672) on 30 November 2025. This study was performed in accordance with the standards of the 1964 Declaration of Helsinki and its later amendments.

### 2.6. Data Analysis

The baseline demographic and clinical characteristics were assessed using the chi-square test or Fisher’s exact test for the categorical variables by documenting the frequencies and percentages. The one-way ANOVA was used for continuous variables by reporting mean ± standard deviation (SD). In contrast, the Kruskal–Wallis test was used for the non-normally distributed variables by reporting the median and interquartile range (IQR). The normality was checked by running the Shapiro–Wilk test. The total number of each drug class was calculated and presented as frequency and percentages. Before running the regression model, the variance inflation factor (VIF) was low among variables, indicating no multicollinearity issues. Thus, multinomial logistic regression with a generalized logit link was applied to identify any associated factors between the outcome variables (no polypharmacy (reference group) vs. polypharmacy and hyperpolypharmacy) and the associated factors such as age, gender, nationality, HbA1c, eGFR, and comorbidities. The odds ratio (OR), along with the 95% confidence interval, was reported. The *p*-value < 0.05 was set as statistically significant. SAS software (version 9.4; SAS Institute Inc., Cary, NC, USA) was used to analyze the data.

## 3. Results

The total number of patients diagnosed with T2DM was 2435. This was represented as patients with no polypharmacy [n = 700 (28.75%)], polypharmacy [n = 1144 (46.98%)], and hyperpolypharmacy [n = 591 (24.27%)]. The mean (SD) age of patients with no polypharmacy was 55.12 (15.14), while polypharmacy was 60.83 (11.73), and hyperpolypharmacy was 63.39 (10.19), with statistically significant differences across all three groups (*p*-value < 0.0001).

The medians (IQR) of HbA1c, albumin, and serum creatinine ranged between 6.60 and 7.70 (5.80–9.10), 4.20 and 4.30 (4.00–4.60), and 0.80 and 0.90 (0.71–1.21), respectively. In addition, the medians (IQR) of eGFR for patients with no polypharmacy were 96.46 (81.99–105.69), polypharmacy 89.09 (71.58–100.09), and hyperpolypharmacy 80.47 (56.61–95.87), which showed a significant difference (*p*-value < 0.0001). The total number of males was 1135, while the number of females was 1300 across all three groups (*p*-value = 0.8501). Most of the participants in our study were Saudis (2042), and non-Saudis were 393, with a *p*-value of 0.0430.

The numbers and percentages of patients diagnosed with HTN with no polypharmacy (n = 49 (17.19%)), polypharmacy (n = 127 (44.56%)), and hyperpolypharmacy (n = 109 (38.25%)) show a significant difference (*p*-value < 0.0001). Additionally, the total number of patients diagnosed with mental disorder and CHF was 103 and 64, respectively. Patients with pulmonary disease were 101 (*p*-value = 0.0110), and those with neurological disease were 36 (*p*-value = 0.0200). In contrast, individuals with cancer were 56, which was not significant (*p*-value = 0.0659). For more details, see Table 1.

The multinomial logistic regression indicates that older age was significantly associated with a higher risk of polypharmacy [OR = 1.031, 95% (1.022–1.040)] and hyperpolypharmacy [OR = 1.037, 95% (1.026–1.049)] compared to no polypharmacy. Patients with higher levels of HbA1c showed a significantly higher risk of polypharmacy and hyperpolypharmacy ([OR = 1.162, 95% (1.105–1.221)] and [OR = 1.284, 95% (1.209–1.364)], respectively). Additionally, eGFR levels represent significantly lower odds of hyperpolypharmacy [OR = 0.985, 95% (0.980–0.991)] than individuals without polypharmacy. Saudi patients were at a significantly lower risk of hyperpolypharmacy [OR = 0.657, 95% (0.478–0.902)], and cancer patients were at a significantly lower risk of polypharmacy [OR = 0.465, 95% (0.242–0.894)] than those without a cancer diagnosis. The risk of polypharmacy and hyperpolypharmacy among individuals diagnosed with mental disorders was significantly higher compared to those without mental disorders [OR = 2.265, 95% (1.206–4.254)] and [OR = 6.269; 95% (3.284–11.964], respectively. The risk of hyperpolypharmacy was reported for patients with HTN [OR = 2.136, 95% (1.449–3.148)], pulmonary disease [OR = 2.375, 95% (1.292–4.367)], and CHF [OR = 8.014, 95% (2.768–23.200)] compared to those without these diseases. More information can be found in Table 2.

The most frequent use of drug classes of patients diagnosed with T2DM were oral antidiabetics 4965 (21.89%) and injectable antidiabetics 4419 (19.48%). The utilization of lipid-lowering agents, proton pump inhibitors, and antiplatelet drugs represents 2868 (12.65%), 1503 (6.63%), and 1297 (5.72%), respectively. In addition, the use of non-steroidal anti-inflammatory drugs was around 855 (3.77%), and calcium channel blockers were 843 (3.72%). On the other hand, the drug classes that represent less than 50 uses (0.22%) were antiparkinsonians, antithyroids, aromatase inhibitors, benzodiazepines, central alpha 2 agonists, antiarrhythmics, and cholinesterase inhibitors. Table 3 shows more information.

## 4. Discussion

In our study, we explored factors that may increase the risk of polypharmacy and hyperpolypharmacy among people diagnosed with T2DM in a single center in Saudi Arabia. The covariates that may play a significant role in polypharmacy and hyperpolypharmacy were older age, people with a mental disorder, and higher levels of A1c and albumin, while eGFR and cancer showed lower odds of a polypharmacy rate. On the other hand, individuals with comorbidities such as HTN, pulmonary disease, and CHF were at higher risk of hyperpolypharmacy. The highest prescribed medication classes were oral antidiabetics, injectable antidiabetics, and lipid-lowering agents. In contrast, the lowest use of drug classes was benzodiazepines, central alpha-2 agonists, antiarrhythmics, and cholinesterase inhibitors.

In our study, the use of five drugs or more among individuals diagnosed with T2DM was dramatically higher, representing 71.25%. These findings were in line with a study conducted in the Riyadh region in 2018, which reported 78% [11]. In addition, the prevalence was 55.2% in Egypt and 83.1% in Oman, which is consistent with our results [20,21]. In other international countries, the polypharmacy rate was represented in the United States of America (57%), Ethiopia (59.1%), China (72.2%), and Denmark (89%) [14,22,23,24]. The prevalence of hyperpolypharmacy in our study was 24.7%, which is nearly aligned with a study in an academic medical center in the Netherlands that reported 29% [25]. Overall, the prevalence rate of using five medications or more was observed to be relatively high in T2DM. Therefore, this could be due to the complexity of T2DM and coexisting complications that may lead to this higher rate [26,27].

In this study, there were significant factors that may influence the rate of polypharmacy and hyperpolypharmacy in patients with T2DM. The older individuals diagnosed with T2DM were at relatively high risk of polypharmacy and hyperpolypharmacy, which is in line with previous studies [11,23,25]. The HbA1c level was a significant predictor that was associated with higher odds of polypharmacy and hyperpolypharmacy in our study, which aligns with a previous study [25]. In contrast, eGFR levels showed a lower significant risk of hyperpolypharmacy, which is consistent with a published study in the literature [25]. This increase could be suspected because the healthcare providers may prescribe protective therapy [28]. Therefore, this underscores the importance of the pharmacist’s role in providing effective medication regimens in the primary care settings [29]. This could be managed by establishing medication therapy management services to avoid the risk of polypharmacy and to control the HbA1C levels [30,31].

In our study, the coexistence of chronic comorbidities with T2DM increases the risk of polypharmacy and hyperpolypharmacy. In the literature, there are multiple studies reporting a high risk of polypharmacy in patients with HTN and/or pulmonary disease, which is not in line with our findings [11,32,33]. However, in our study, patients diagnosed with HTN and/or pulmonary disease have twice the risk of using 10 or more drugs. In addition, those with CHF were 8 times more at risk of hyperpolypharmacy but not of polypharmacy. Therefore, there were studies in the literature that found a high risk of polypharmacy in patients with DM and HF [10,34]. The definition of polypharmacy did not consist between the studies, even though individuals were at high risk of using multiple medications due to the complexity of the two diseases [35]. This increase in cardiovascular diseases could be due to the clinical guidelines that recommended prescribing a certain type of drug for treatment and prevention [36]. Additionally, the pathophysiological complex of the cardiovascular-kidney syndrome (CKM) could lead to polypharmacy [37]. The cardiorenal protection drugs are highly recommended for patients with T2DM to minimize the risk of kidney diseases [38]. Thus, the role of the healthcare providers should be to review the patients’ medications at each visit and reduce the inappropriate drugs [39]. In addition, there should be clinical strategies for deprescribing unnecessary drugs and encouraging a lifestyle to improve patients’ outcomes [40]. Individuals with documented mental health disease and T2DM in our study were at a higher risk of being prescribed five or more medications, which is consistent with published studies in the literature [11]. Consequently, the high number of drug users could be explained by the association of mental health disease and T2DM [41].

In our study, the highest prescribed number of medications was antidiabetics, then lipid-lowering agents, which aligned with previous studies [23,25]. In addition, there were a high number of drugs that were prescribed for patients with HTN, such as calcium channel blockers, beta blockers, and angiotensin receptor blockers. The reason for this explanation is that there were patients in our study present with coexisting T2DM and HTN. In contrast, one of the least used drug classes in our study was benzodiazepines, which is consistent with a published study [24].

The healthcare providers should be aware of the healthcare expenditures and complications of patients with T2DM [42]. In addition, providing effective patient care strategies could lead to minimizing related costs, hospitalizations, and polypharmacy [43,44]. Therefore, this could be achieved by offering a comprehensive drug review to manage patients’ drug therapy by establishing an ambulatory care clinic led by a clinical pharmacist [45]. This study has several strengths. First, this is the first study in Saudi Arabia that assessed polypharmacy along with hyperpolypharmacy in a large sample size for our study. Second, we used electronic medical records that contain comprehensive data such as lab values. Third, we were able to calculate the eGFR for each patient based on the CKD-EPI formula. However, there were some limitations in our study. First, the cross-sectional design limits the causal relationship between polypharmacy, hyperpolypharmacy, and comorbidities. Second, we did not specify indications for each medication because our goal was to assess the number of drugs, whether appropriately or inappropriately prescribed. Finally, our results limited generalizability to other regions in Saudi Arabia.

## 5. Conclusions

The prevalence of polypharmacy and hyperpolypharmacy is high in patients with T2DM. In addition, the predictors that may play a significant role in increasing the risk of polypharmacy and/or hyperpolypharmacy were the poor control of HbA1c and eGFR levels. Additionally, the other coexisting chronic comorbidities of T2DM patients were HTN, mental disorder, pulmonary disease, and CHF. Providing proper prescribing and managing patients’ therapy plans can improve individuals’ health outcomes. Therefore, this highlights the important role of primary care physicians in coordinating the care, along with clinical pharmacists, in the prevention and identification of polypharmacy.

## Figures and Tables

**Table 1:** Baseline characteristics of the number of medications used among patients with type 2 diabetes mellitus.

Variable	No Polypharmacy <5 [n = 700 (28.75%)]	Polypharmacy 5 to 9 [n = 1144 (46.98%)]	Hyperpolypharmacy ≥10 [n = 591 (24.27%)]	*p*-Value *
**Age in Years—mean (SD)**	55.12 (15.14)	60.83 (11.73)	63.39 (10.19)	<0.0001
**Hemoglobin A1C (%)—median (IQR)**	6.60 (5.80–7.60)	7.30 (6.40–8.50)	7.70 (6.70–9.10)	<0.0001
**Albumin (g/dL)—median (IQR)**	4.30 (4.10–4.60)	4.30 (4.10–4.50)	4.20 (4.00–4.50)	<0.0001
**Serum creatinine (mg/dL)—median (IQR)**	0.80 (0.71–0.94)	0.84 (0.73–1.05)	0.90 (0.76–1.21)	<0.0001
**eGFR (ml/min/1.73 m^2^)—median (IQR)**	96.46 (81.99–105.69)	89.09 (71.58–100.09)	80.47 (56.61–95.87)	<0.0001
**Gender**				
Male	324 (28.55%)	540 (47.58%)	271 (23.88%)	0.8501
Female	376 (28.92%)	604 (46.46%)	320 (24.62%)	
**Nationally**				
Saudi	597 (29.24%)	969 (47.45%)	476 (23.31%)	0.0430
Non-Saudi	103 (26.21%)	175 (44.53%)	115 (29.26%)	
**Hypertension**				
Yes	49 (17.19%)	127 (44.56%)	109 (38.25%)	<0.0001
No	651 (30.28%)	1017 (47.30%)	482 (22.42%)	
**Cancer**				
Yes	21 (37.50%)	18 (32.14%)	17 (30.36%)	0.0659
No	679 (28.54%)	1126 (47.33%)	574 (24.13%)	
**Mental Disorders**				
Yes	14 (13.59%)	41 (39.81%)	48 (46.60%)	<0.0001
No	686 (29.42%)	1103 (47.30%)	543 (23.28%)	
**Pulmonary Diseases**				
Yes	19 (18.81%)	46 (45.54%)	36 (35.64%)	0.0110
No	681 (29.18%)	1098 (47.04%)	555 (23.78%)	
**Neurological Disease**				
Yes	4 (11.11%)	18 (50.00%)	14 (38.89%)	0.0200
No	696 (29.01%)	1126 (46.94%)	577 (24.05%)	
**Congestive Heart Failure**				
Yes	4 (6.25%)	20 (31.25%)	40 (62.50%)	<0.0001
No	696 (29.35%)	1124 (47.41%)	551 (23.24%)	
**Dyslipidemia**				
Yes	12 (21.05%)	28 (49.12%)	17 (29.82%)	0.3479
No	688 (28.93%)	1116 (46.93%)	574 (24.14%)	

*p*-value < 0.05 *.

**Table 2:** Multinomial logistic regression of the number of medications among patients with type 2 diabetes mellitus.

Variables	No Polypharmacy (Ref.)					
	Polypharmacy			Hyperpolypharmacy		
	Odds Ratio	95% CI		Odds Ratio	95% CI	
Age *	1.031	1.022	1.040	1.037	1.026	1.049
**Female**	0.969	0.797	1.180	1.020	0.804	1.295
**HbA1c**	1.162	1.105	1.221	1.284	1.209	1.364
**eGFR ^†^**	0.996	0.991	1.001	0.985	0.980	0.991
**Saudi**	0.896	0.681	1.179	0.657	0.478	0.902
**Hypertension**	1.350	0.941	1.937	2.136	1.449	3.148
**Cancer**	0.465	0.242	0.894	0.900	0.446	1.816
**Mental**	2.265	1.206	4.254	6.269	3.284	11.964
**Pulmonary**	1.572	0.898	2.752	2.375	1.292	4.367
**Neurological**	1.319	0.436	3.985	1.475	0.464	4.688
**Congestive Heart Failure**	2.383	0.802	7.085	8.014	2.768	23.200
**Dyslipidemia**	1.333	0.660	2.695	1.468	0.654	3.296

* Age (per 1-year increase); ^†^ eGFR (per 1 mL/min/1.73 m^2^ increase).

**Table 3:** The most commonly prescribed medication among patients with type 2 diabetes mellitus.

Class	Number	Percent
**Oral Antidiabetics**	4965	21.89
**Injectable Antidiabetics**	4419	19.48
**Lipid-Lowering Agents**	2868	12.65
**Proton Pump Inhibitors**	1503	6.63
**Antiplatelets**	1297	5.72
**Non-Steroidal Anti-Inflammatory Drugs**	855	3.77
**Calcium Channel Blockers**	843	3.72
**Beta Blockers**	769	3.39
**Thyroid Agents**	748	3.3
**Angiotensin Receptor Blockers (ARBs)**	668	2.95
**ARB and Thiazide Diuretic Combinations**	590	2.6
**Anticonvulsants**	557	2.46
**Antidepressants**	424	1.87
**Topical Corticosteroids**	392	1.73
**Angiotensin-Converting Enzyme (ACE) Inhibitors**	374	1.65
**Diuretics**	346	1.53
**Antipsychotics**	163	0.72
**Systemic Corticosteroids**	155	0.68
**Anticoagulants**	149	0.66
**Antiangiogenics**	126	0.56
**Antigout**	112	0.49
**Angiotensin Receptor-Neprilysin Inhibitor (ARNI)**	82	0.36
**Vasodilators**	66	0.29
**Antiosteoporosis**	53	0.23
**Antiparkinsonians**	44	0.19
**Antithyroids**	38	0.17
**Aromatase Inhibitors**	21	0.09
**Benzodiazepines**	16	0.07
**Central Alpha 2 Agonist**	16	0.07
**Antiarrhythmics**	12	0.05
**Cholinesterase Inhibitors**	9	0.04
**Total**	**22,680**	**100.00**

## Data Availability

The data used in this study are available upon reasonable request from the corresponding author (Mohammed M. Alsultan).

## Abbreviations

The following abbreviations are used in this manuscript:

T2DM	Type 2 diabetes mellitus
DM	Diabetes mellitus
HTN	Hypertension
CHF	Congestive heart failure
SD	Standard deviation
MENA	Middle East and North Africa
EMRs	Electronic medical records
HbA1c	Hemoglobin A1c
eGFR	Glomerular filtration rate
IQR	Interquartile range
OR	Odds ratio
CKD-EPI	Chronic kidney disease epidemiology
